# Modeling the Amplification Dynamics of Human *Alu* Retrotransposons

**DOI:** 10.1371/journal.pcbi.0010044

**Published:** 2005-09-30

**Authors:** Dale J Hedges, Richard Cordaux, Jinchuan Xing, David J Witherspoon, Alan R Rogers, Lynn B Jorde, Mark A Batzer

**Affiliations:** 1 Department of Biological Sciences, Biological Computation and Visualization Center, Center for Bio-Modular Microsystems, Louisiana State University, Baton Rouge, Louisiana, United States of America; 2 Department of Human Genetics*,* University of Utah Health Sciences Center*,* Salt Lake City, Utah, United States of America; 3 Department of Anthropology, University of Utah, Salt Lake City, Utah, United States of America; Pennsylvania State University, United States of America

## Abstract

Retrotransposons have had a considerable impact on the overall architecture of the human genome. Currently, there are three lineages of retrotransposons (*Alu,* L1, and SVA) that are believed to be actively replicating in humans. While estimates of their copy number, sequence diversity, and levels of insertion polymorphism can readily be obtained from existing genomic sequence data and population sampling, a detailed understanding of the temporal pattern of retrotransposon amplification remains elusive. Here we pose the question of whether, using genomic sequence and population frequency data from extant taxa, one can adequately reconstruct historical amplification patterns. To this end, we developed a computer simulation that incorporates several known aspects of primate *Alu* retrotransposon biology and accommodates sampling effects resulting from the methods by which mobile elements are typically discovered and characterized. By modeling a number of amplification scenarios and comparing simulation-generated expectations to empirical data gathered from existing *Alu* subfamilies, we were able to statistically reject a number of amplification scenarios for individual subfamilies, including that of a rapid expansion or explosion of *Alu* amplification at the time of human–chimpanzee divergence.

## Synopsis

Nearly 50% of the human genome is composed of mobile elements. While much of this sequence consists of inactive “fossil” elements that are no longer actively moving or generating new copies, three families are currently proliferating in human genomes. Among these, the *Alu* lineage has reached a copy number of over 1 million and alone accounts for approximately 10% of the genome. While considerable evidence has been gathered concerning the underlying biological mechanisms of *Alu* mobilization and proliferation, a detailed understanding of *Alu* amplification history is currently lacking. Researchers are aware, for example, that several thousand *Alu* elements have inserted within the human genome since the divergence of humans and chimpanzees, but how those insertions were distributed over this ~6-million-year time period is currently unknown. In this work, the authors introduce a simulation framework that seeks to incorporate both sequence diversity and empirically gathered population data from human *Alu* elements, in order to provide a better understanding of the last several million years of human *Alu* evolution. The results suggest that a rapid explosion of *Alu* amplification at the time of the human–chimpanzee divergence is unlikely. Therefore, it is improbable that an increase in *Alu* retrotransposition activity was involved in the speciation of humans and chimpanzees.

## Introduction

A collection of evolutionarily recent and older “fossil” mobile element sequences compose more than 45% of the human genome [1–5]. Along with the recently characterized SVA family, *Alu* and L1 have the distinction of being the only mobile element lineages to be actively proliferating in modern humans [3,6,7]. All three of these lineages belong to the retrotransposon class of mobile elements, replicating themselves via an RNA intermediate [6,8]. They differ, however, in that L1 retrotransposons are ~6-kb-long autonomous elements that encode the proteins required for their retrotransposition [2] while *Alu* and SVA retrotransposons are shorter, non-autonomous elements that are *trans*-mobilized by the L1 protein machinery [9]. L1 elements have been active in mammalian genomes for the past 150 million years (myrs) and have reached a copy number of ~0.5 million in the human genome, and *Alu* retrotransposons have reached a copy number of ~1.1 million within the past 65 myrs [1]. By comparison, the SVA lineage is a relative newcomer to the primate lineage, having achieved a copy number of approximately 5,000 copies in the human genome over the last 15 myrs [7]. Together, the amplification activity of these retrotransposon families has had a substantial impact on their host genomes. In addition to contributing to genome size expansion, they have shaped the architecture of the human genome by mediating genetic exchanges such as duplications, deletions, inversions, transductions, and translocations [6,8,10–17]. L1 and *Alu* have also been implicated in DNA repair [18] and alteration of gene expression [2,19–21]. As they are still actively retrotransposing and thus acting as insertional mutagens, *Alu,* SVA, and L1 elements are responsible for more than 0.5% of all human genetic disorders [2,22,23].

While much attention has been given to the underlying biology driving retrotransposon expansion in primates, little attempt has been made to assess what can broadly be described as “amplification dynamics.” Under this heading we include the evolutionary window during which lineages were actively retrotransposing, the intensity of retrotransposition, and the degree of rate fluctuation during this period. Notable exceptions to this general dearth of information concerning mobile element amplification dynamics include data for mobile element activity in *Drosophila* species [24–26]. While a considerable body of theoretical work exists concerning mobile element expansion (e.g., [27–33]), these models generally focus on element copy number behavior under equilibrium conditions and do not address the impact of diverse amplification histories on sequence composition. The observation of divergent mobile element retrotransposition levels among closely related host species [24,34], however, suggests that the assumption of equilibrium may often be unrealistic, as noted in [35]. A more complete understanding of how mobile element sequence structure and frequencies are influenced by diverse nonequilibrium expansion scenarios would be invaluable for developing realistic models of how transposable elements spread through given taxa.

The problem we are faced with is how to reconstruct the evolutionary amplification history of a mobile element lineage given only a static snapshot of sequence and polymorphism data from present-day genomes. Previously, efforts used the phylogenetic distribution of mobile element lineages to bound their period of activity in time by the divergence dates of their host taxa (e.g., [36–38]). While such analyses can provide useful information, particularly where allele frequency information is unavailable, they nevertheless cannot yield the kind of temporal resolution that would be most helpful in understanding the amplification process. For example, we know that some 6,000–7,000 *Alu* elements have fixed in the human genome since *Pan troglodytes* and *Homo sapiens* last shared a common ancestor 5–8 myrs ago [39–41], but the temporal pattern of expansion giving rise to these elements remains unknown. Age estimates of individual retrotransposon insertions based on sequence divergence from a consensus typically possess a great degree of uncertainty because of the relatively short sequence lengths of many retrotransposons, particularly among short interspersed elements, as well as because of uncertainty over the accuracy of the consensus “source” sequence used for comparison [42–45]. In younger, recently active retrotransposon lineages, an additional piece of evidence is at our disposal to aid in reconstructing their amplification history. For these families, we are able to obtain population frequency data for insertions at given loci, which allow estimation of the percentage of polymorphic loci for presence/absence in the corresponding subfamilies (termed in the following text “insertion polymorphism level” [IPL]).

Alone, sequence diversity and IPL prove insufficient to reconstruct the historical amplification pattern of a mobile element family with any degree of accuracy. When effectively combined, however, we hypothesized that they may serve to narrow the alternative scenarios. We tested this hypothesis by focusing on the *Alu* family of retrotransposons, for which subfamily structure is well characterized and population frequency data are available for a number of distinct subfamilies [3,39–41,46]. Furthermore, *Alu* retrotransposons presented an attractive target for this initial study because, as detailed below, they possess several features that make the process of modeling their retrotransposition more tractable. It was first necessary to determine what set of *Alu* sequence and IPL observations might be expected under various evolutionary amplification patterns. To generate quantitative expectations for these parameters under diverse patterns of expansion, we developed a computer simulation that incorporates established aspects of *Alu* retrotransposon biology (see Materials and Methods). Our simulation also accommodates the effect of significant sampling biases inherent in the way *Alu* elements have been characterized in the human genome. By comparing existing *Alu* sequence diversity and polymorphism levels, we were able to statistically reject multiple amplification scenarios for individual *Alu* subfamilies, resulting in a more refined understanding of the retrotransposition dynamics of human-specific *Alu* subfamilies.

## Results/Discussion

### The *Alu* Simulation Framework

Two fundamental processes underlie the various descriptive statistics that can be tabulated from genomic *Alu* sequences, namely the post-insertion evolution of *Alu* nucleotide sequences and the associated evolution of insertion polymorphism allele frequencies. To make the modeling process more straightforward, we divided these processes into distinct core simulator programs, one to model the behavior of nucleotide sequence and one to model the behavior of inserted retrotransposon allele frequencies. Several of the known properties of *Alu* subfamily structure and sequence mutation patterns were incorporated within the programs (see Materials and Methods). Both programs implement a strict “master gene” model of *Alu* retrotransposition under which a single source element produces inert, non-retrotransposing copies [47]. While it has been demonstrated that most *Alu* subfamilies deviate from the strict master gene model, this scenario can nevertheless explain the majority of overall subfamily sequence structure [48]. More importantly, implementing deviations from the master gene model (i.e., secondary and tertiary retrotransposition sources and so on) can lead to exponential copy number increase when limiting factors such as negative selection do not constrain numbers, a scenario which is clearly not historically accurate. In this analysis, we have restricted our simulation to neutrally evolving loci within a panmictic population of constant size. In our model we also assume that retrotransposition rates (RRs) do not fluctuate during the window of time during which retrotransposition occurs.

The above assumptions are almost certainly oversimplifications, but are necessary to keep the number of amplification scenarios at a manageable level in this initial investigation. We believe the existence of secondary source genes would have a limited impact on our analysis because any secondary source that is active enough to produce appreciable copy numbers would be classified as a separate subfamily under current naming conventions, and it would be analyzed separately in our approach. The effect of population substructure is more difficult to anticipate because the nature of population substructure during the time period in question is largely unknown. Significant population structure would impact the behavior of polymorphisms by extending their average persistence time, affecting the rate of insertion polymorphism decay both during and after transposition. The nature and magnitude of these effects will be the subject of future investigations.

For both sequence mutation and frequency drift simulations, retrotransposition started at time *t*_0_ and proceeded at a constant rate for a time window *T*_retro_. Thus, given a subfamily copy number *n, T*_retro_ defines the RR of the simulation (i.e., RR = *n*/*T*_retro_). For the sequence mutation simulations, elements were allowed to mutate neutrally from their initial time of retrotransposition until the end of the simulation. *T*_mut_ represents the total elapsed simulation time, which is also the amount of time the oldest element in the subfamily has been evolving. We have chosen a maximum *T*_mut_ of 6 myrs, as this roughly corresponds to the human–chimpanzee divergence time and, thus, is suitable for investigating the amplification dynamics of recent human *Alu* subfamilies. During the course of each run, sequence variation and allele frequency statistics (described in detail below) were calculated at 100,000-y observation intervals. One thousand replicates were performed under each of seven basic amplification models (M0 to M6), ranging from M0, which has an instantaneous burst of insertion activity generating all subfamily members, to M6, in which new retrotransposition events occur at a uniform rate from the beginning of the subfamily throughout the entire simulation of 6 myrs. Intermediate models (M1 to M5), in which amplification occurred for 1 to 5 myrs and then ceased, were also evaluated. Simulations were performed using a human effective population size (*N*_e_) of 10,000 individuals and a generation time of 25 y. To assess the impact of alternative values for *N*_e_ and generation time, we also performed simulations using *N*_e_ values of 5,000, 15,000, and 20,000 individuals as well as generation times of 20 and 30 y.

### Amplification History and Sequence Variation

As an estimator of *Alu* subfamily sequence variation, we used the parameter π, which is defined as the mean number of nucleotide differences observed among all pairs of *Alu* sequences in the subfamily [49]. For example, a π value of three means that there are, on average, three nucleotide differences between any two *Alu* sequences in the subfamily. Means, modes, and standard deviations for π were calculated across all replicates (available at http://batzerlab.lsu.edu). In addition to π, we evaluated the use of the mismatch distribution raggedness index as a metric of sequence diversity [50], but its informativeness proved limited for our purposes, and it was excluded from subsequent analyses.

**Figure 1:**
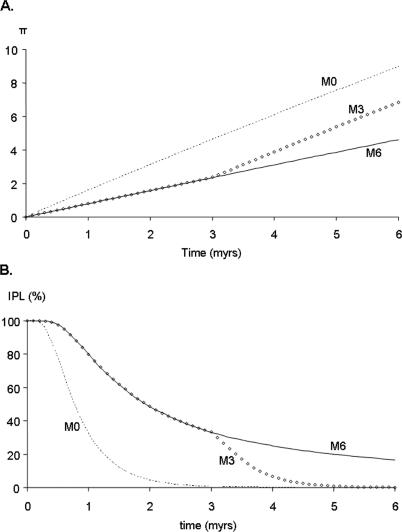
Temporal Variation of Subfamily Sequence Variation π and IPL Results for three expansion models are shown, in which retrotransposition activity was instantaneous (M0) or lasted for 3 (M3) or 6 (M6) myrs. Variation in π (A) is slowed during retrotransposition, but increases immediately upon the cessation of retrotransposition. Rate of IPL decay (B) is attenuated during retrotransposition activity but increases once retrotransposition ends.

As expected, mean π values increased linearly with time in our simulation (Figure 1A). The effect of retrotransposition during *T*_retro_ is to slow the rate of increase in π. In scenarios M1 through M6, where retrotransposition occurs for a period of time then ceases, the rate of π increase becomes steeper (though still linear) immediately following the cessation of retrotransposition (Figure 1A). A clear relationship exists between sequence diversity and the particular amplification model of the family. For example, a scenario with a burst of retrotransposition followed by dormancy leads to higher π values than scenarios where RR has been uniform over long periods of time. This result is intuitive, as any scenario resulting in an increased element insertion number earlier in a subfamily's history will result in additional opportunity for mutation and consequently higher π values. The problem, however, is that when evaluating real mobile element data, the time of onset of retrotransposition (i.e., the beginning of *T*_retro_) is typically unknown. From examining Figure 1A, it is evident that any value of π can be obtained by any model, provided that an appropriate amount of time (*T*_mut_) has elapsed prior to the point of observation. Thus, although π is directly influenced by the particular amplification history, it cannot be used to infer that history without additional information.

### *Alu* Insertion Polymorphism

In addition to π, we also modeled the behavior of IPL, which indicates the percentage of polymorphic insertion loci in a subfamily. Like π, IPL is expected to be influenced by both the age of the subfamily and its historical pattern of retrotransposition. Figure 1B illustrates the decay of IPL over time under models M0, M3, and M6. As might be expected, ongoing retrotransposition in a mobile element family slows the rate of IPL decay by providing an influx of new polymorphisms. When retrotransposition ceases, IPL falls relatively rapidly over the course of approximately 1 myrs. This rate of IPL breakdown is consistent with the expected on average 1-myr coalescence time (4*N*_e_ generations, where *N*_e_ is the effective population size) for our simulated human effective population size of 10,000 individuals. As with π, there is clearly an effect of amplification history on IPL values, with IPL values remaining higher for those families whose *T*_retro_ windows extended closer to the present day. But also, as is the case with π, any scenario can yield a given IPL value depending on what time point (*T*_mut_ value) is being sampled. A researcher examining empirical *Alu* frequency data does not know what position his or her data occupy on the timeline of the model of retrotransposition being considered (Figure 1B). Yet, as we demonstrate below, for a given model there exists a set of IPL and π parameters that are mutually exclusive across a range of time points. As a consequence, by combining the π and IPL statistics, one can effectively narrow the possible range of amplification histories for a given *Alu* subfamily.

### Inferring Amplification Scenarios from Genomic *Alu* Data

**Figure 2:**
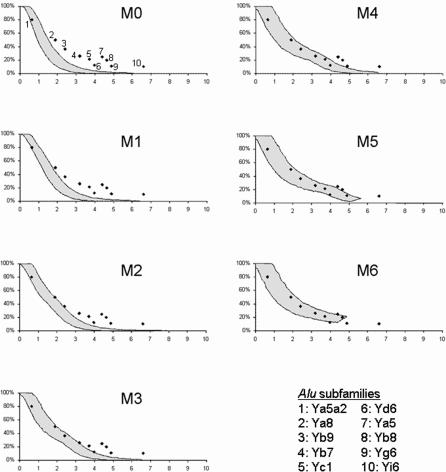
Distribution of Subfamily Sequence Variation π (*x*-Axis) versus IPL (*y*-Axis) Expectations based on 1,000 replicates of expansion models M0–M6. Shaded area in each plot indicates 95% of resulting values for each model. Observed (π and IPL) values for ten recent human *Alu* subfamilies are shown as black diamonds. These results are based on a generation time of 25 y and an effective population size of 10,000 individuals.

Plots of IPL versus π for equivalent time points over the course of seven amplification scenarios (i.e., models M0–M6) are shown in Figure 2, based on a generation time of 25 y and an effective population size of 10,000 individuals. The 95% confidence intervals, generated from 1,000 simulation replicates, are represented as the bounded area in each graph (see Materials and Methods). In addition, π values were estimated for ten human *Alu* subfamilies for which IPL data are available (Table 1). These data were collected from subsets of elements from the respective polymorphic subfamilies for which population data were available. For each of these subfamilies, the relationship between IPL and π is indicated in Figure 2. In our analysis, if a subfamily's IPL versus π point falls within the 95% confidence interval of a given model's results, the model cannot be excluded as a possible amplification pattern (see Materials and Methods for details). Conversely, when a subfamily's data point falls outside the bounded area, that model can be excluded for the subfamily in question.

**Table 1:**
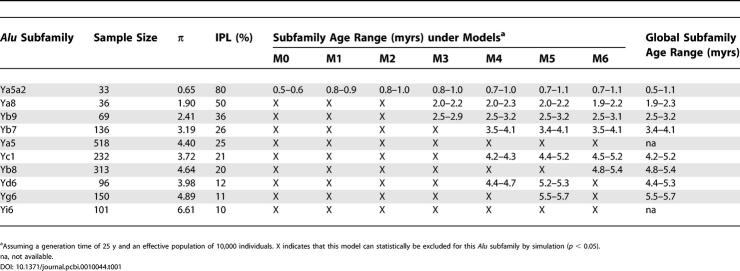
*Alu* Subfamily Diversity (π) and IPL Parameters and Their Age under Different Models of Amplification

### Impact of Effective Population Size and Generation Time Parameters

Our initial simulation replicates were conducted under the conditions of a 25-y generation time and effective population size of 10,000 breeding individuals. While these represent commonly accepted values for these parameters, we also investigated the impact of a broader range of generation times (20 and 30 y) and *N*_e_ (5,000, 15,000, and 20,000) on the simulations. For *N*_e_ = 5,000, our models fail to encompass most of the observed data for extant *Alu* subfamilies (Figure S1; Table S1). This is not unexpected, as this *N*_e_ value is approximately half that of most literature estimates. Likewise, *N*_e_ = 20,000 yields IPL and π values that are largely not concordant with observed *Alu* subfamily data (Figure S2; Table S1). *N*_e_ values of 10,000 and 15,000 individuals manage to encompass the majority of observed *Alu* data points (Figures 2 and S3; Tables 1 and S1). In this respect, the behavior of our simulations is congruent with current literature estimates, which place the human *N*_e_ on the order of 10,000 to 15,000 individuals [51–53].

Altering the generation time also had an appreciable effect on simulation behavior by shifting the timescale of the simulated data. While a generation time of 20 y did not perform very well (Figure S4; Table S2), our models were generally able to encompass more observed *Alu* subfamily data under generation time parameters of 25 and 30 y (Figures 2 and S5; Tables 1 and S2). Such values lie within the range of currently estimated values for ancestral generation times spanning the relevant period ([54] and references therein). Also, as discussed below, a higher generation time parameter of 30 y has the effect of bringing *Alu* subfamily age estimates derived from our simulation closer in line with previous literature values determined by other methods.

### Estimating the Age of *Alu* Subfamilies

**Figure 3:**
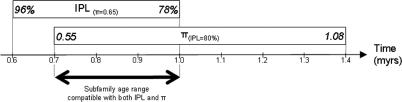
Estimation of the Age of the Ya5a2 *Alu* Subfamily under Simulation M4 In M4, *N*_e_ is 10,000 and generation time is 25 y. Data are based on observed subfamily sequence variation π and IPL parameters. Time estimates consistent with π and IPL values are indicated in boxes. The bold double arrow indicates age estimates concordant with both parameters.

Once improbable amplification scenarios are excluded (Tables 1, S1, and S2), it is possible to determine time periods of amplification for subfamilies that are compatible with both their π and IPL values. By using the present time as a point of reference (i.e., *T*_mut_ = present), it is further possible to infer the age of the subfamilies. Figure 3 illustrates this process. In this example, the Ya5a2 subfamily has a π value (0.65) that is consistent with an age ranging from 0.6 to 1.0 myrs before present under M4 (*N*_e_ = 10,000, generation time = 25 y). Within that range, the Ya5a2 IPL value is only compatible with 0.7 to 1.0 myrs before present. Estimated age ranges that are consistent with both π and IPL for all *Alu* subfamilies analyzed in this study under a generation time of 25 y and *N*_e_ of 10,000 are given in Table 1. We note here that *Alu* subfamily age estimates derived in this study are generally higher than those reported in previous literature [42]. However, the age estimates obtained from sequence diversity alone typically have large standard deviations [42] that overlap with our estimates derived from both sequence diversity and IPL. This might indicate that time estimates derived from sequence diversity alone may underestimate the true ages of the subfamilies. Nevertheless, alternative values of *N*_e_ and generation time also have an impact on the potential age of the subfamily as estimated by our method. For example, when age calculations are made using a generation time of 30 y, our age estimates more closely approximate those of previous literature.

Our results suggest that, while a range of retrotransposition scenarios remain possible for each subfamily, some alternatives can be statistically rejected. Notably, when using standard values for effective population size (*N*_e_ =10,000) and generation time (25 y), our results exclude the possibility that the majority of human *Alu* insertions occurred during a brief, intense burst of retrotransposition activity after the divergence between humans and chimpanzees. Such a scenario, intermediate between M1 and M0 (instantaneous) results in an IPL versus π distribution well outside the observed data points (see Figure 2). This result is well supported because variation in effective population size and generation time leads to the same conclusion (Figures S1–S5). Thus, these analyses provide evidence against the notion of a burst of retrotransposition at or near the human–chimpanzee divergence. This result is consistent with a previous study [34], which suggested that the marked increase in human *Alu* fixation events with respect to chimpanzee was initiated within the past 4 myrs. The involvement of mobile element amplification activity in the formation of reproductive barriers, and hence speciation, has received a fair amount of attention [55–58], although definitive evidence is lacking. The discovery of high levels of mobile element activity in humans compared to chimpanzees [34,59] has invited speculation as to whether or not the *Alu* retrotransposition increase might have been involved in the speciation of humans and chimpanzees [59]. While our present results do not support an increase in mobile element activity at the time of the human–chimpanzee divergence, they do not exclude the possibility of such an event during a later hominid speciation event. Furthermore, the possibility remains that an extended simulation model, one that accounts for additional biological and spatial parameters, may generate results that are consistent with a retrotransposon burst at the time of speciation.

### Conclusion

We have demonstrated that it is possible to mine information concerning the amplification history of a retrotransposon subfamily from present-day genomic and population data. Overall, there appears to be heterogeneity in both the timing and intensity of human *Alu* subfamily activity. Our simulations do not presently accommodate the influence of host population subdivision, RR fluctuations (i.e., rate heterogeneity) over time, and selection on patterns of retrotransposon evolution. All of these phenomena will likely have some bearing on the nucleotide divergence and IPL, although the extent of that influence is difficult to anticipate. We plan to extend our simulations to encompass these and other potentially relevant phenomena in further studies. Nevertheless, the present analyses do indicate that the combination of retrotransposon sequence divergence and subfamily polymorphism information has the potential to reveal information about the historical dynamics of mobile element amplification that has thus far remained inaccessible. In particular, by applying our method we are able to rigorously address the issue of the time window during which amplification occurred. A more detailed account of the history of retrotransposon activity will allow for a better understanding of the forces that influence mobile element activity across diverse taxa.

## Materials and Methods

### Simulating *Alu* sequence evolution.

We developed a simulator of *Alu* sequence evolution that takes into account most of the major known properties of *Alu* elements in terms of subfamily structure and sequence mutation patterns. Specifically, *Alu* elements begin accumulating nucleotide substitutions stochastically, starting at the time of retrotransposition and until the end of the simulation. Nucleotide substitution was simulated using the Kimura two-parameter reversible mutation model, a neutral mutation rate at non-CpG dinucleotides of 0.0015 mutations/site/myrs [60] and a transition to transversion ratio of four. To accommodate the known mutation bias for *Alu* CpG dinucleotides as a result of the deamination of methylated cytosines, CpG dinucleotides were allowed to mutate at a 6-fold higher rate than non-CpG dinucleotides [42]. To make the modeling process more computationally tractable, we assumed a scenario of *Alu* subfamily evolution in which *Alu* retrotransposition followed a strict master gene model, with a lone, non-mutating source sequence generating offspring that were incapable of additional retrotransposition. We also considered the *Alu* expansion to have occurred in a single, representative genome, with each successful retrotransposition event equivalent to a “substitution” event at the population level. This allowed for combining *Alu* retrotransposition events with standard methods for calculating substitution probabilities, greatly reducing simulation complexity and computational time.

### Decay of IPLs.

To study the evolution of IPL during the transposition process, we modeled the behavior of IPL under the same model conditions as π (i.e., M0 through M6, as described above). Given the low probability of fixation for each initial insertion event (1/2*N*_e_), several million retrotransposition events must ultimately be followed in order to achieve final subfamily copy numbers comparable to those observed in the human genome. In each model, 7 million insertion events occurring over various windows of time were used to yield approximately 350 fixed elements. To reduce computational time, Kimura's recursion approximation of the diffusion process was used to simulate the neutral drift of retrotransposed elements [61]. The absorption boundaries [0,1] at which alleles were lost or fixed, respectively, were adjusted slightly to compensate for disparities between the continuous results from the recursion equation and the discrete frequencies that real-world alleles can assume. (The continuous values between zero and 1/2*N*_e_ are possible return values from the recursion, but not realistic allele frequencies.) A generation time of 25 y and effective population size of 10,000 interbreeding individuals was used. To address uncertainty surrounding ancestral human generation times and effective population sizes, the effects of a range of generation times (20, 25, and 30 y) and effective population sizes (5,000, 10,000, 15,000, and 20,000) were investigated. At the onset of the simulation, the number of retrotranspositions per time increment required to achieve the 7 million insertion target was calculated. Allele frequencies were allowed to drift randomly both during and after transposition windows, and IPL values were calculated and reported at 100,000-y intervals.

### Accounting for IPL sampling effects.

To adequately model the element copy number and IPL values observed in the human genome, the manner in which genomic elements are ascertained and characterized was also incorporated into the simulation. The population sample size from which most *Alu* elements have so far been initially discovered is effectively a single individual (i.e., the human genome draft sequence), and, consequently, a considerable number of polymorphic elements will remain unobserved. When simulating the observed IPL value, the effect of ascertaining elements from a single individual must be accommodated. In order to do so, the number of polymorphic elements that were reported as “observed” at any given time during the simulation was determined by effectively sampling a single individual from the simulated population. In this step, the detection of a given *Alu* insertion polymorphism within that individual was stochastically determined, with the probability of observing a given insertion being proportional to the frequency of the insertion in the population. The simulations were implemented in a set of C language programs with assisting Perl scripts and are available at http://batzerlab.lsu.edu.

### Statistical evaluation of models.

Models were excluded or not excluded based on 95% confidence intervals generated through simulation. For each model scenario (M0 to M6), 1,000 replicates were simulated. IPL and π values were calculated at 100,000-y intervals for the simulated datasets, and the lower and upper 1.265 percentiles were used to determine the 95% confidence interval. Boundary values for 95% confidence interval were adjusted for the effect of two independent tests of the IPL and π parameters resulting from the model. Here, the probability of falling outside the range of some percentage, *X,* of the simulated data twice (two tests) is given by 1 – (1 − *X*)^2^. To determine the boundaries that would be appropriate at the 5% significance level, we solved the equation 1 – (1 − *X*)^2^ = 0.05, yielding *X* = 0.0253. Upper and lower boundaries were then 0.0253/2 = 0.01265. π versus IPL values for real *Alu* subfamilies were then plotted together with the simulated data. If a given subfamily's π versus IPL data fell outside the 95% confidence interval of a given model, the model was rejected for that subfamily.

### Evaluating the impact of subfamily size.

**Figure 4:**
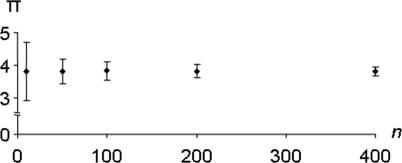
Impact of Subfamily Copy Number *(n)* on the Sequence Variation π Parameter Increasing subfamily size beyond 100 copies had little effect on between-replicate variation.

All the analyses above were conducted using subfamily copy numbers of approximately 350 elements for the nucleotide evolution simulation and 7 million insertion events (corresponding to ~350 fixations) for IPL modeling. To assess the impact of subfamily size on the behavior of π, we simulated sequence evolution for *T*_mut_ = 2 myrs in an *Alu* subfamily having generated *n* = 50, 100, 200, and 400 copies under a retrotransposition model where all elements were produced at *t*_0_ (i.e., *T*_retro_ = 0). We performed 100 simulation replicates for each value of *n*. We found that the major effect of increasing *n* was to decrease the standard deviation of π among trials, but otherwise copy number had little impact on the behavior of π over time (Figure 4). The reduction of between-trial variance due to increasing family size stabilized at copy numbers greater than 100 elements. We therefore ran the all simulations described above using *n* = 350 elements, a number that is in the same order of magnitude of size as most of the observed *Alu* subfamilies used in our study. Similar tests were conducted for IPL simulations using alternate insertion numbers (1 million, 5 million, and 10 million). While some subfamilies in the study, namely Ya5 and Yb8, are considerably larger than 350 in observed copy number, experimentation with copy numbers as high as 5,000 demonstrate that higher subfamily sizes reduces between-replicate variance (data not shown).

## Supporting Information

Figure S1 Distribution of Subfamily Sequence Variation π (*x*-Axis) versus IPL (*y*-Axis): Generation Time of 25 y and *N*_e_ of 5,000 Individuals Expectations based on 1,000 replicates of expansion models M0–M6. The two lines indicate the boundaries of the 95% confidence interval for each model. Observed (π and IPL) values for ten recent human *Alu* subfamilies are shown as black diamonds (see legend of Figure 2).
(3325 KB TIF)

Figure S2 Distribution of Subfamily Sequence Variation π (*x*-Axis) versus IPL (*y*-Axis): Generation Time of 25 y and *N*_e_ of 20,000 Individuals Expectations based on 1,000 replicates of expansion models M0–M6. The two lines indicate the boundaries of the 95% confidence interval for each model. Observed (π and IPL) values for ten recent human *Alu* subfamilies are shown as black diamonds (see legend of Figure 2).
(3.3 MB TIF)

Figure S3 Distribution of Subfamily Sequence Variation π (*x*-Axis) versus IPL (*y*-Axis): Generation Time of 25 y and *N*_e_ of 15,000 Individuals Expectations based on 1,000 replicates of expansion models M0–M6. The two lines indicate the boundaries of the 95% confidence interval for each model. Observed (π and IPL) values for ten recent human *Alu* subfamilies are shown as black diamonds (see legend of Figure 2).
(3.3 MB TIF)

Figure S4 Distribution of Subfamily Sequence Variation π (*x*-Axis) versus IPL (*y*-Axis): Generation Time of 20 y and *N*_e_ of 10,000 Individuals Expectations based on 1,000 replicates of expansion models M0–M6. The two lines indicate the boundaries of the 95% confidence interval for each model. Observed (π and IPL) values for ten recent human *Alu* subfamilies are shown as black diamonds (see legend of Figure 2).
(3.4 MB TIF)

Figure S5 Distribution of Subfamily Sequence Variation π (*x*-Axis) versus IPL (*y*-Axis): Generation Time of 30 y and *N*_e_ of 10,000 Individuals Expectations based on 1,000 replicates of expansion models M0–M6. The two lines indicate the boundaries of the 95% confidence interval for each model. Observed (π and IPL) values for ten recent human *Alu* subfamilies are shown as black diamonds (see legend of Figure 2).
(3.4 MB TIF)

Table S1 *Alu* Subfamily Compatibility with Different Retrotransposition Models (M0–M6) for Different Effective Population Sizes (*N*_e_) and a Generation Time of 25 y (72 KB DOC)

Table S2 *Alu* Subfamily Compatibility with Different Retrotransposition Models (M0–M6) for Different Generation Times and an Effective Population Size of 10,000 Individuals (56 KB DOC)

## Abbreviations

IPL: insertion polymorphism level
M[number]: model [number]
myrs: million years
RR: retrotransposition rate
