# Supplementary figures and images for: Modeling the Amplification Dynamics of Human Alu Retrotransposons
Source: PLoS Comput Biol. 2005 Sep 30;1(4):e44. doi: 10.1371/journal.pcbi.0010044 (PMC1239904; doi:10.1371/journal.pcbi.0010044)

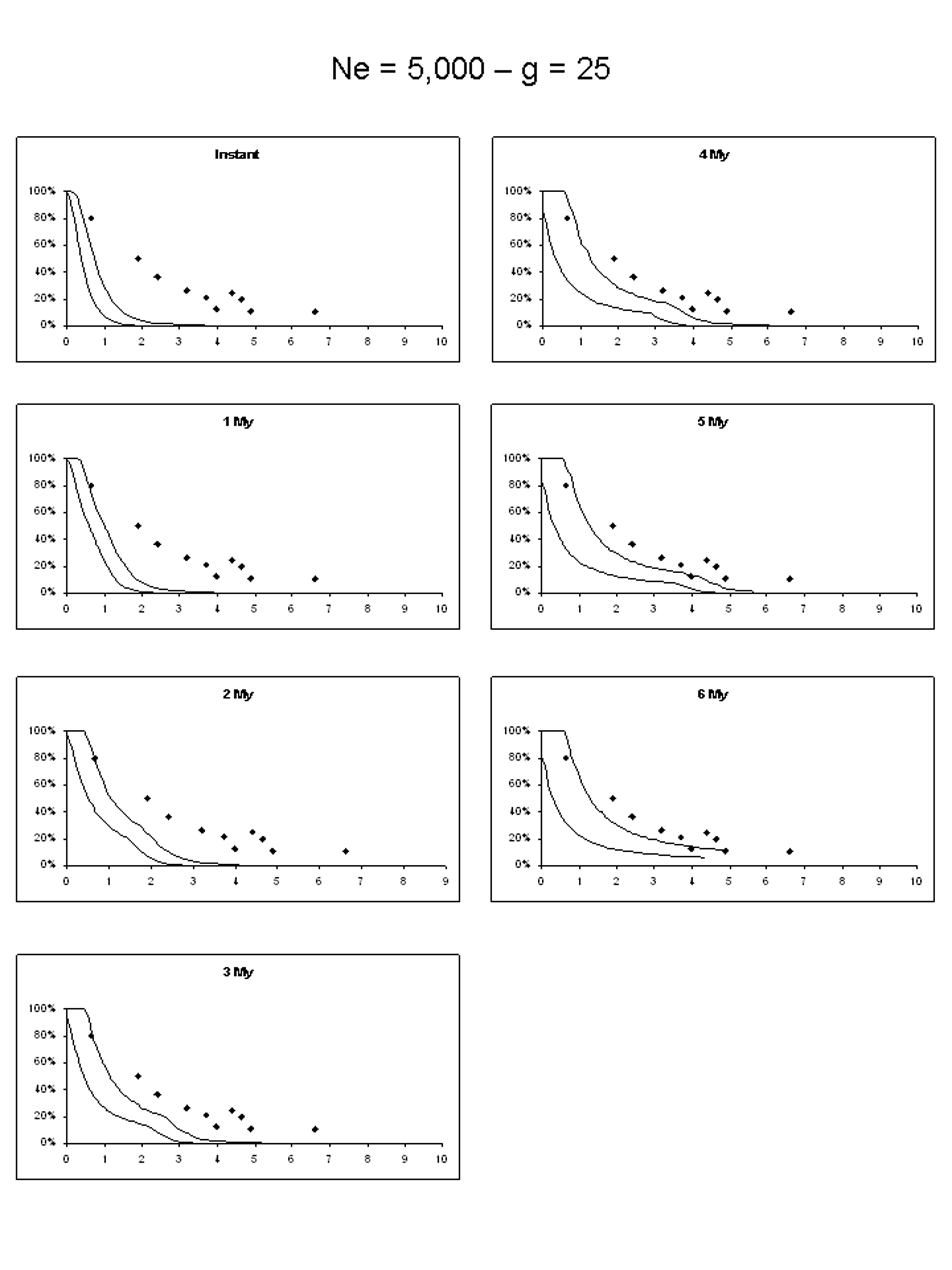

Supplement: Figure S1 — Expectations based on 1,000 replicates of expansion models M0–M6. The two lines indicate the boundaries of the 95% confidence interval for each model. Observed (π and IPL) values for ten recent human Alu subfamilies are shown as black diamonds (see legend of Figure 2). (3325 KB TIF) [file pcbi.0010044.sg001.tif]

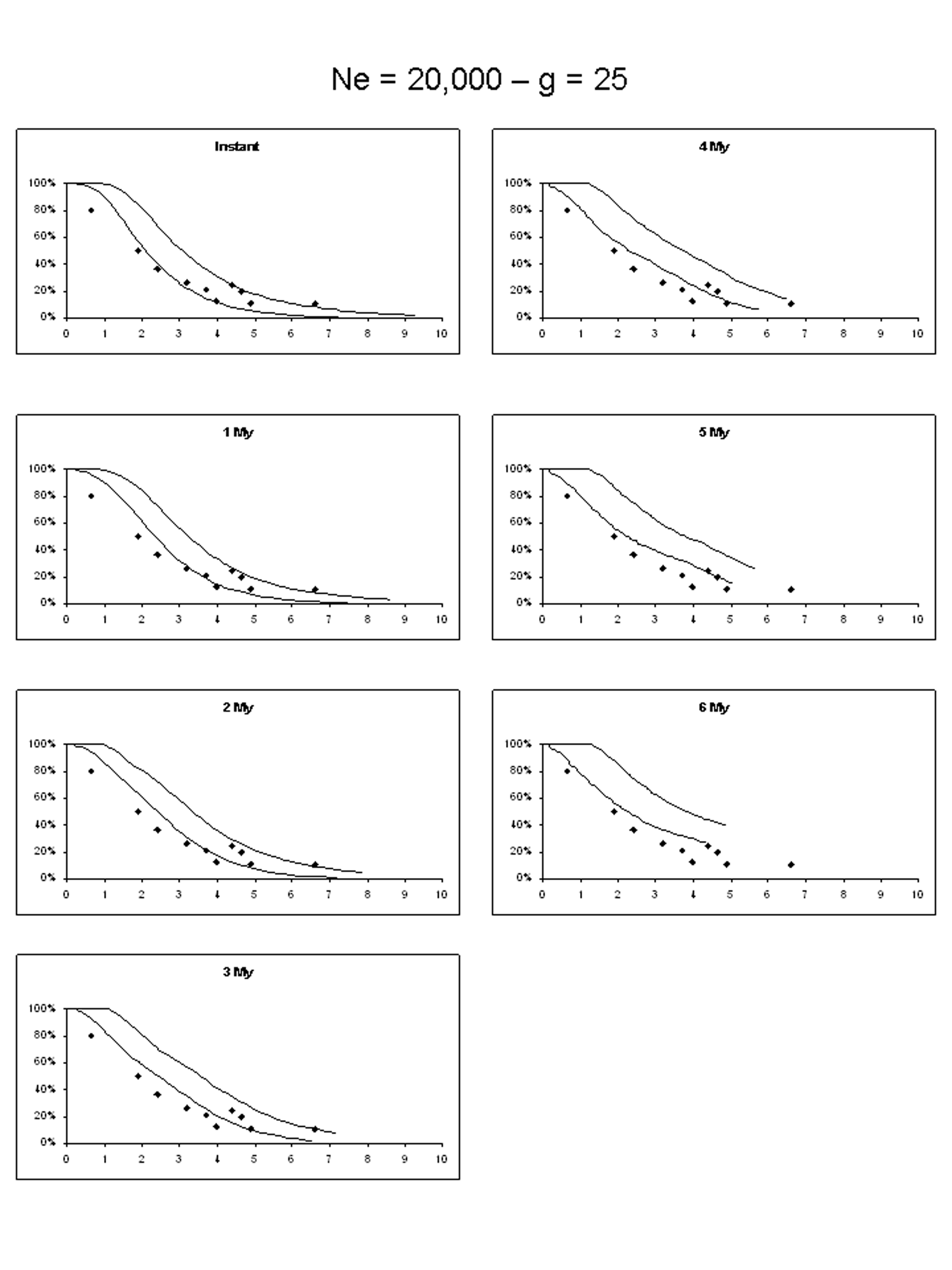

Supplement: Figure S2 — Expectations based on 1,000 replicates of expansion models M0–M6. The two lines indicate the boundaries of the 95% confidence interval for each model. Observed (π and IPL) values for ten recent human Alu subfamilies are shown as black diamonds (see legend of Figure 2). (3.3 MB TIF) [file pcbi.0010044.sg002.tif]

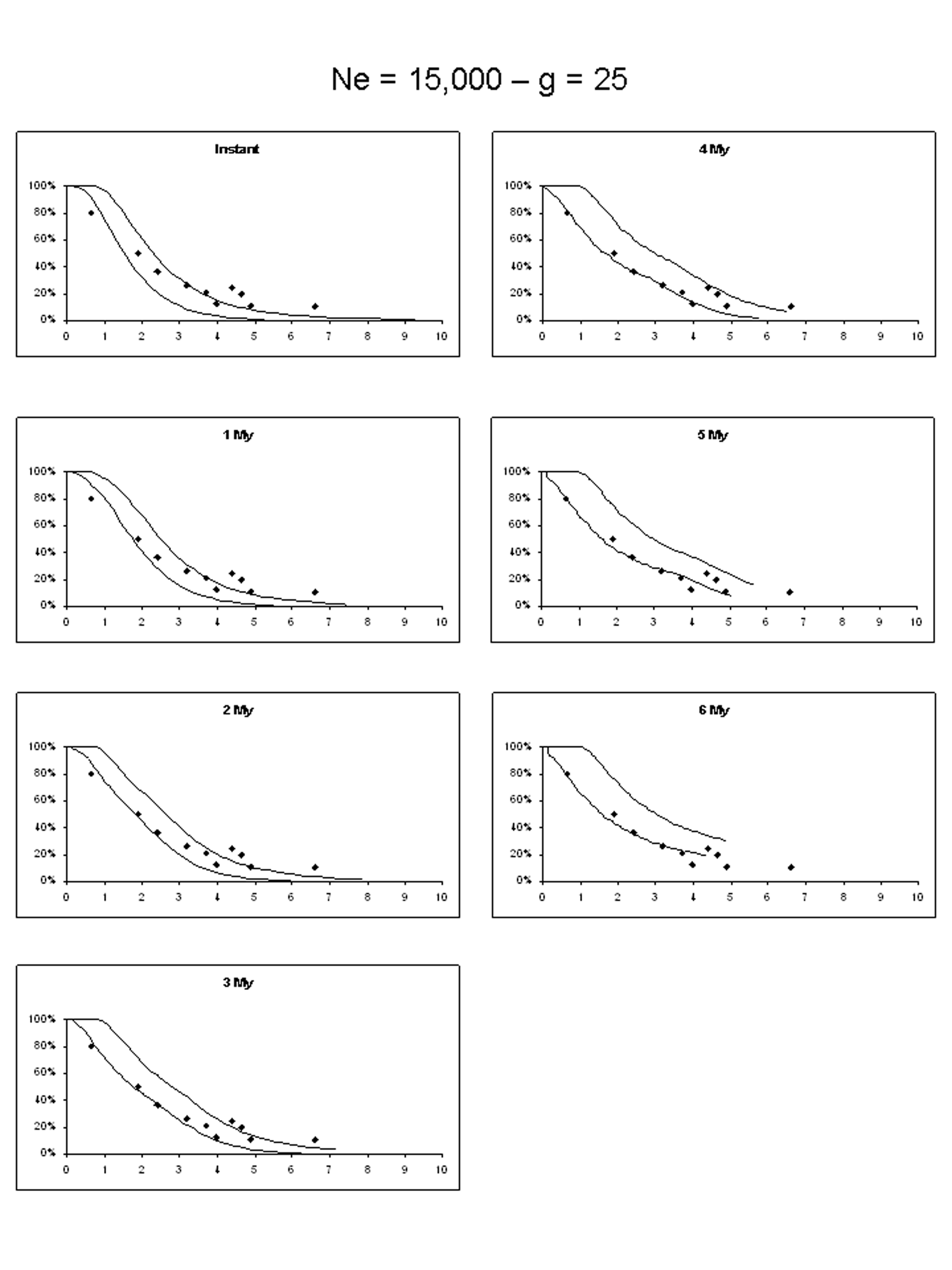

Supplement: Figure S3 — Expectations based on 1,000 replicates of expansion models M0–M6. The two lines indicate the boundaries of the 95% confidence interval for each model. Observed (π and IPL) values for ten recent human Alu subfamilies are shown as black diamonds (see legend of Figure 2). (3.3 MB TIF) [file pcbi.0010044.sg003.tif]

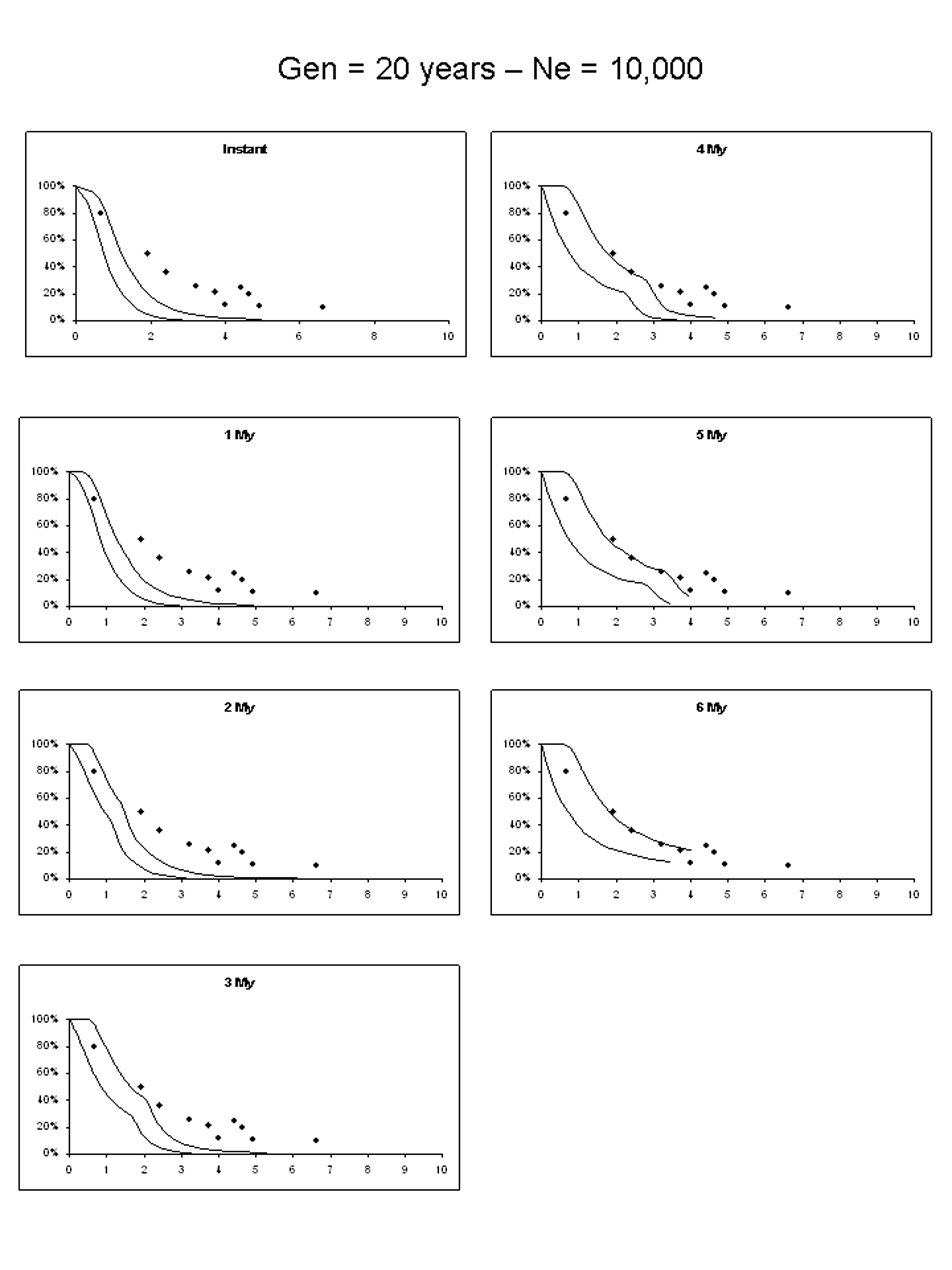

Supplement: Figure S4 — Expectations based on 1,000 replicates of expansion models M0–M6. The two lines indicate the boundaries of the 95% confidence interval for each model. Observed (π and IPL) values for ten recent human Alu subfamilies are shown as black diamonds (see legend of Figure 2). (3.4 MB TIF) [file pcbi.0010044.sg004.tif]

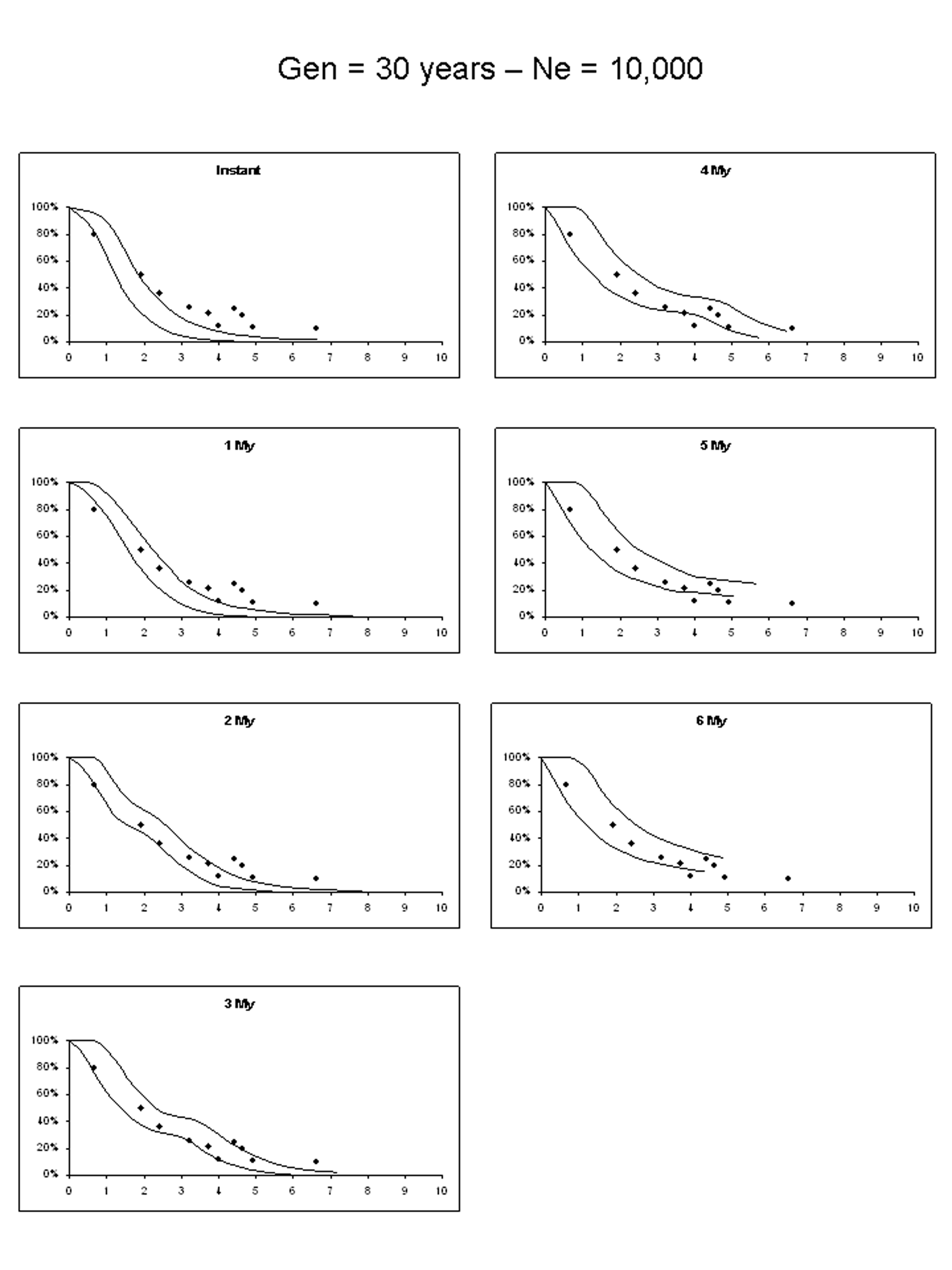

Supplement: Figure S5 — Expectations based on 1,000 replicates of expansion models M0–M6. The two lines indicate the boundaries of the 95% confidence interval for each model. Observed (π and IPL) values for ten recent human Alu subfamilies are shown as black diamonds (see legend of Figure 2). (3.4 MB TIF) [file pcbi.0010044.sg005.tif]
