# Supplementary material for: Modeling the Amplification Dynamics of Human Alu Retrotransposons
Source: PLoS Comput Biol. 2005 Sep 30;1(4):e44. doi: 10.1371/journal.pcbi.0010044 (PMC1239904; doi:10.1371/journal.pcbi.0010044)
Supplement: Table S1 — (72 KB DOC) [file pcbi.0010044.st001.doc]

**Table S1.** *Alu* subfamily compatibility with different retrotransposition models (M0-M6) for different effective population sizes (N_e_) and a generation time of 25 years.

| Model | Instant | 1 My | 2 My | 3 My | 4 My | 5 My | 6 My |
| --- | --- | --- | --- | --- | --- | --- | --- |
| Ne=5,000 |  |  |  |  |  |  |  |
| Ya5a2 | X | X | O | O | O | O | O |
| Ya8 | X | X | X | X | X | X | X |
| Yb9 | X | X | X | X | X | X | X |
| Yb7 | X | X | X | X | X | X | X |
| Ya5 | X | X | X | X | X | X | X |
| Yc1 | X | X | X | X | X | X | X |
| Yb8 | X | X | X | X | X | X | X |
| Yd6 | X | X | X | X | X | O | O |
| Yg6 | X | X | X | X | X | X | X |
| Yi6 | X | X | X | X | X | X | X |
|  |  |  |  |  |  |  |  |
| Ne=15,000 |  |  |  |  |  |  |  |
| Ya5a2 | X | X | X | X | X | X | O |
| Ya8 | O | O | O | O | O | O | O |
| Yb9 | O | O | O | X | X | O | O |
| Yb7 | O | O | O | O | O | X | X |
| Ya5 | X | X | X | X | O | O | O |
| Yc1 | X | O | O | O | O | X | X |
| Yb8 | X | X | X | X | O | O | X |
| Yd6 | O | O | O | O | X | X | X |
| Yg6 | X | X | X | O | O | O | X |
| Yi6 | X | X | X | X | X | X | X |
|  |  |  |  |  |  |  |  |
| Ne=20,000 |  |  |  |  |  |  |  |
| Ya5a2 | X | X | X | X | X | X | X |
| Ya8 | X | X | X | X | X | X | X |
| Yb9 | X | X | X | X | X | X | X |
| Yb7 | O | X | X | X | X | X | X |
| Ya5 | X | O | O | O | O | O | X |
| Yc1 | O | O | X | X | X | X | X |
| Yb8 | O | O | O | O | O | O | X |
| Yd6 | X | X | X | X | X | X | X |
| Yg6 | O | O | O | O | X | X | X |
| Yi6 | X | X | X | O | X | X | X |

Note: X = rejected model

O = compatible model
